# Assessing the Impact of the Prone Position on Acute Kidney Injury

**DOI:** 10.3390/jcm14020631

**Published:** 2025-01-19

**Authors:** Eden Ezra, Itai Hazan, Dana Braiman, Rachel Gaufberg, Jonathan Taylor, Adva Alyagon, Amit Shira Babievb, Lior Fuchs

**Affiliations:** 1 Joyce and Irving Goldman Medical School, The Faculty of Health Sciences, Ben-Gurion University of the Negev, Beer-Sheva 8410501, Israel; itaihazan@gmail.com (I.H.); danabra@clalit.org.il (D.B.); adva.alyagon@gmail.com (A.A.); liorfuchs@gmail.com (L.F.); 2 Clinical Research Center, Soroka University Medical Center, Faculty of Health Sciences, Ben-Gurion University of the Negev, Beer-Sheva 8410501, Israel; amitsba@clalit.org.il; 3 Medical Intensive Care Unit, Soroka University Medical Center, Beer-Sheva 8410501, Israel; 4 Department of Pediatrics, Yale School of Medicine, New Haven, CT 06510, USA; gaufberg@post.bgu.ac.il; 5 Interdepartmenal Division of Critical Care Medicine, University of Toronto, Toronto, ON M5S 1A1, Canada; jonathan.taylor2303@gmail.com

**Keywords:** acute kidney injury (AKI), acute respiratory distress syndrome (ARDS), body mass index (BMI), obesity, prone position

## Abstract

**Background**: Prone positioning is a standard intervention in managing patients with severe acute respiratory distress syndrome (ARDS) and is known to improve oxygenation. However, its effects on other organs, particularly the kidneys, are less well understood. This study aimed to assess the association between prone positioning and the development of acute kidney injury (AKI), specifically in overweight and obese patients. **Methods**: A retrospective pre–post study was conducted on a cohort of 60 critically ill ARDS patients who were placed in the prone position during hospitalization. The development of AKI was assessed using the Acute Kidney Injury Network (AKIN) criteria, with AKI measured by both creatinine levels (AKIN_Cr_) and urine output (AKIN_UO_). Patients were divided into two groups based on body mass index (BMI): overweight/obese (BMI ≥ 25) and non-obese (BMI < 25). Data were collected before and after prone positioning. **Results**: In overweight/obese patients (n = 39, 57 cases), both the median AKIN_Cr_ and AKIN_UO_ scores increased significantly following prone positioning (from 0 to 1, median *p* < 0.01, and from 0 to 2, median *p* < 0.01, respectively). No statistically significant changes in AKIN scores were observed in non-obese patients nor were significant differences found in either group after repositioning to supine. **Conclusions**: Prone positioning is associated with an increased risk of acute kidney injury in overweight and obese ARDS patients. This may be due to the kidneys’ susceptibility to intra-abdominal hypertension in these patients. Further research is needed to explore optimal proning strategies for overweight and obese populations.

## 1. Introduction

Acute respiratory distress syndrome (ARDS) is a clinical condition characterized by acute, diffuse inflammatory lung injury leading to respiratory failure, affecting approximately 10% of all intensive care unit (ICU) admissions [1]. Current ARDS management guidelines recommend prone positioning as a therapeutic intervention, showing a significant mortality benefit [2]. The landmark PROSEVA randomized controlled trial demonstrated that prone positioning for at least 16 h per day in patients with moderate-to-severe ARDS significantly reduced both 28-day and 90-day mortality, as well as the incidence of cardiac arrests [3]. Prone positioning improves oxygenation by reducing intrapulmonary shunting, decreasing ventilation–perfusion mismatch, and preventing ventilator-induced lung injury by distributing mechanical stress and strain more evenly throughout the lung tissue [4].

The COVID-19 pandemic has drawn further attention to this treatment approach, leading to a global increase in proning practices. COVID-19 patients with ARDS who were placed in the prone position showed significant improvements in oxygenation [5,6], with benefits comparable to those seen in non-COVID-19 ARDS patients, even in non-ICU settings [7]. Much of the benefit from prone positioning is attributed to the redistribution of the weight of the heart and abdominal organs away from the lungs, reducing atelectasis [8].

Gong et al. previously reported a positive association between body mass index (BMI) and the risk of developing ARDS [9]. The global rise in obesity prevalence, along with the increasing frequency of ICU admissions for obese patients, makes this association a critical area of ARDS research [10,11,12]. Obese patients tend to experience greater morbidity during ICU admission [12,13,14]. Outcomes in ARDS are significantly worsened when extra-pulmonary multi-organ failure develops, particularly renal failure [15]. The association between acute kidney injury (AKI) and poor outcomes is well documented [16,17]. Furthermore, patients who develop AKI during ICU admission have a significantly increased risk of long-term mortality, extending to at least two years post discharge [18]. Similar to ARDS, the incidence of AKI is substantially higher among critically ill obese patients [19,20,21,22].

Known risks associated with prone positioning include hemodynamic changes, hypoperfusion, ophthalmic injury, and compartment syndrome [23]. Several studies have explored the potential association between prone positioning and organ failure [24,25,26]; however, no definitive conclusions have been reached regarding the relationship between prone positioning and the development of renal failure.

Given the established risks of both ARDS and AKI, particularly in obese patients, we aimed to determine whether prone positioning in ARDS patients is associated with the development of AKI.

## 2. Materials and Methods

### 2.1. Study Population

This retrospective pre–post study design used each patient as their own control, comparing data before (“pre proning”) and after (“post proning”) the intervention. The intervention was defined as changing the patient’s position from supine to prone or vice versa. This study was conducted at Soroka University Medical Center (SUMC), a tertiary care academic medical center in southern Israel. The cohort included all patients with moderate-to-severe ARDS who were admitted to the ICU between January 2010 and October 2021, all of whom were under mechanical ventilation and underwent prone positioning during their hospital stay. ARDS was diagnosed based on the Berlin criteria [27]. This study was approved by the Institutional Review Board (IRB) (protocol code SCRC20017) with a waiver of informed consent.

The criteria for prone positioning followed ARDS guidelines, as established by the PROSEVA trial (*p* ratio < 150), but decisions regarding whether to prone a patient were made in real time at the treating physician’s discretion. Patients were excluded from the analysis if they received any diuretic drugs within six hours before or after proning or if they had renal failure requiring renal replacement therapy (RRT). This exclusion criterion applied both to patients with chronic end-stage renal disease on dialysis prior to their ARDS diagnosis and those with acute renal failure requiring new RRT. Both continuous forms of RRT (e.g., continuous veno-venous hemodiafiltration) and conventional hemodialysis were considered exclusionary forms of RRT.

### 2.2. Data Collection

We screened the institutional electronic medical records to identify ICU patients hospitalized during the study period with an ICD-9 diagnosis of ARDS who were placed in the prone position at least once during their hospital stay. Data were manually extracted through chart review. Collected variables included the following:

- Patient demographics: gender, age, comorbidities, and BMI.
- ICU-related data: length of stay, vital signs, vasopressor requirements, concurrent medications, use of diuretics, hourly urine output, and levels of creatinine, urea, and lactate.

Measurements were recorded 24 h before and 24 h after a change in patient position, either from supine to prone or from prone back to supine. Baseline creatinine was defined as the lowest value recorded during the year prior to proning. If no previous records were available, the lowest creatinine value from the current hospitalization was used.

The severity of illness was assessed using the Sequential Organ Failure Assessment (SOFA) score [28]. In addition, pre- and post-proning blood pressure, vasopressor dose, and lactate levels were collected to account for potential hemodynamic confounders that could contribute to AKI development.

AKI severity was measured 24 h before and after intervention using the Acute Kidney Injury Network (AKIN) classification, which categorizes AKI into three stages based on changes in serum creatinine and urine output:

- Stage 1: serum creatinine increase of at least 1.5 times baseline or absolute increase of 0.3 mg/dL (AKIN_Cr_ 1) or urine output < 0.5 mL/kg/h for 6–12 h (AKIN_UO_ 1).
- Stage 2: serum creatinine increase of 2.0 times baseline (AKIN_Cr_ 2) or urine output < 0.5 mL/kg/h for over 12 h (AKIN_UO_ 2).
- Stage 3: serum creatinine increase of at least three times baseline, serum creatinine of 4.0 mg/dL or higher (AKIN_Cr_ 3), initiation of renal replacement therapy, urine output < 0.3 mL/kg/h for 24 h, or anuria for 12 h (AKIN_UO_ 3) [29,30,31].

The estimated glomerular filtration rate (eGFR) was then calculated using the Salazar–Corcoran equation, which was developed to estimate the eGFR in obese patients by taking into account the patient’s weight and height [32,33].

### 2.3. Statistical Analysis

Descriptive statistics were used to summarize the demographic and clinical characteristics, baseline laboratory values, and development of AKI before and after a change in patient position. The Wilcoxon signed-rank test was used to compare pre and post continuous variables. Logistic regression was used to assess the association between prone position and AKI development, with odds ratios and 95% confidence intervals reported. AKI development was the dependent variable in the regression model. Statistical significance was set at *p* < 0.05. All analyses were performed using R software.

## 3. Results

### 3.1. General Information

**Figure 1:**
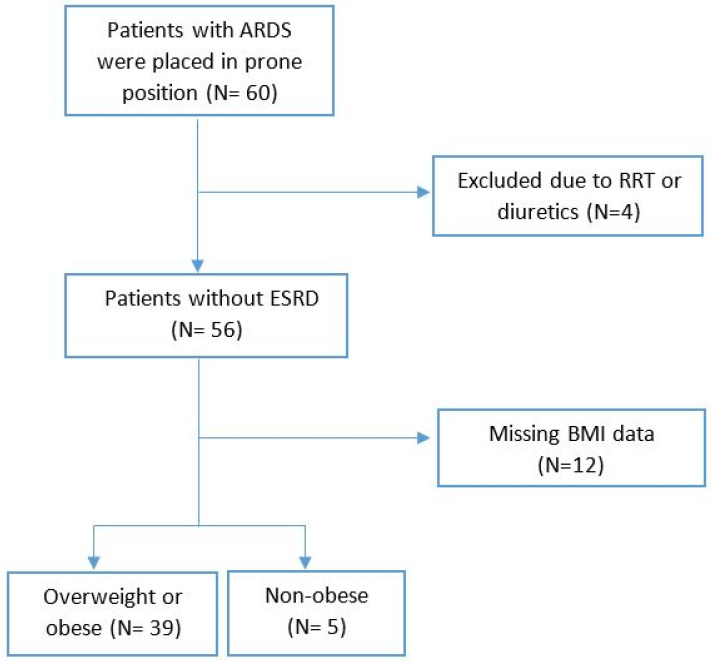
Flow chart for outcome analysis. A total of 56 patients with acute respiratory distress syndrome (ARDS) who were placed in prone position were included in the first analysis, then grouped by body mass index (BMI) level. ESRD = end-stage renal disease; RRT = renal replacement therapy.

A total of 60 patients were admitted to the ICU at SUMC between January 2010 and October 2021 with a diagnosis of moderate-to-severe ARDS and were placed in the prone position during their hospital stay. Patients who underwent RRT within 24 h before or after proning (n = 2), and those who received diuretics within six hours of pronation (n = 2), were excluded from this study. The remaining 56 patients comprised the study population and were included in the analysis (Figure 1). The cohort was predominantly male (71%, n = 40), with a median age of 59 years and a mean BMI of 33 (range 20 to 52). Thirty-nine patients (70%) were diagnosed with COVID-19 infection. The mean baseline creatinine was 0.79 mg/dL, the median PaO_2_/FiO_2_ before proning was 64, and the pre-proning SOFA score had a median of 8 (Table 1). Detailed patient baseline comorbidities are shown in the Supplementary Materials: Table S1. Thirty-nine patients died during their ICU stay, and five additional patients died within six months of ICU discharge. The average ICU length of stay was 27.73 days.

In the cohort of 56 patients, there were 86 distinct supine-to-prone positioning episodes (“proning”) conducted as part of ARDS management. A total of 34% of the cohort (n = 19) required multiple periods in the prone position during their hospital stay. Of the 86 proning episodes, only 59 corresponding supination maneuvers (i.e., returning the patient from prone to supine) were included in the data analysis. Patients were excluded from the supination analysis due to the need for RRT, concurrent diuretic administration, or death while in the prone position, accounting for the discrepancy in the number of proning and supination episodes available for analysis.

The duration of time that patients remained in the prone position varied based on the discretion of the treating physician, with a mean duration of 65 h and 14 min and a median duration of 48 h and 30 min. This extended time in the prone position was attributed to the gradual integration of the PROSEVA trial recommendations, as well as the high proportion of COVID-19 patients in the cohort. The large number of ventilated patients during the COVID-19 pandemic, restrictions on healthcare worker exposure to infected patients, and the low caregiver-to-patient ratio made frequent position changes difficult, preventing adherence to the 16 h goal for supination, as recommended by the PROSEVA study.

Table S2 in the Supplementary Materials presents the laboratory results collected before and after both proning and supination maneuvers, with the most abnormal level measured within 24 h after the change in position reported. The mean lactate level increased significantly after proning, rising from 2.17 mmol/L to 3.24 mmol/L (*p* < 0.01). A similar significant increase was observed following supination, with lactate levels increasing from a mean of 1.78 mmol/L to 2.64 mmol/L (*p* < 0.01). There were no significant changes in the mean arterial pressure (MAP) or vasopressor requirements within 24 h after either pronation or supination. Additionally, the SOFA score did not show any significant changes (Supplementary materials: Table S3).

After pronation maneuvers, the mean creatinine level increased significantly from 0.98 mg/dL to 1.26 mg/dL within 24 h of proning (*p* < 0.01). A similar significant trend was seen in serum urea, which increased from a mean of 73 mg/dL to 86 mg/dL within 24 h of proning (*p* < 0.01). Following supination, no significant change in creatinine was observed (1.09 mg/dL to 1.1 mg/dL, *p* = 0.11). However, serum urea levels did rise significantly after supination, increasing from a mean of 79 mg/dL to 86 mg/dL (Supplementary Materials: Table S4).

### 3.2. AKIN Criteria

As mentioned in the Methods, AKIN scores were calculated for all patients based on creatinine (AKIN_Cr_) and also on urine output (AKIN_UO_) [29,30,31]. In the overall cohort, the mean AKIN_Cr_ exhibited a significant increase from 0.53 before transitioning to the prone position to 0.93 after proning. However, given the ordinal nature of the AKIN score, the median AKIN_Cr_ was also assessed, and no disparities were found, with AKIN 0 (i.e., no AKI) observed both before and after the proning maneuver (measured as worst recorded AKI 24 h post maneuver change, Table 2, *p* < 0.01). Conversely, a different trend emerged in AKIN_UO_ within the overall cohort, with a significant increase from AKIN 0 pre prone to AKIN 1 post prone (Table 3, *p* < 0.01). When patients were placed in a supine position, no statistically significant differences were detected in the AKIN scores, whether based on creatinine or urine output (Table 2 and Table 3).

### 3.3. Obesity Subgroup Analysis

Pre-planned subgroup analyses were conducted on the basis of weight and age, focusing exclusively on pronation. Patients were divided according to BMI into the overweight/obese (BMI ≥ 25) and non-obese (BMI < 25) cohorts (Supplementary Materials: Table S5). Within these subgroups, the overweight or obese group included most of the patients, 57 proning events in thirty-nine patients, while the non-obese group included 11 proning events in five patients (Table 2 and Table 3). Eighteen proning events were missing data regarding patient height or weight and so were not included in the pre-planned subgroup analysis. A separate pre-specified subgroup analysis by age was also performed, separating patients into those below 60 years old and above or equal to 60 to see if the results were stratified by age (Supplementary Materials: Tables S6 and S7).

In the subgroup of overweight or obese patients (n = 39 patients, 57 instances of proning), the median AKIN_Cr_ exhibited a notable increase from AKIN 0 pre prone to AKIN 1 post prone (see Table 2, *p* < 0.01). However, no statistically significant differences were observed in AKIN_Cr_ before and after proning for non-obese patients or for the entire cohort when transitioning from prone to supine (Table 2). A similar trend was observed for the AKIN_UO_ score. Among obese patients, urine output decreased post proning, and the median AKIN_UO_ increased from AKIN 0 to AKIN 2 following proning (median, *p* < 0.01), while no significant changes were noted in the AKIN_UO_ score for non-obese patients (Table 3). The transition from prone to supine did not yield statistically significant alterations in either the AKIN_Cr_ or AKIN_UO_ scores (Table 2 and Table 3).

Additionally, in the obese population, a near-significant reduction in the eGFR was observed following prone positioning, with a mean decrease from 139 to 129 (*p* = 0.07) (Supplementary Materials: Table S8).

### 3.4. Age Subgroup Analysis

A separate pre-planned subgroup analysis by age revealed notable differences. When assessing AKIN_Cr_, a notable increase in mean AKIN_Cr_ was evident, rising from 0.46 to 0.85 in patients under 60 years of age and from 0.63 to 1.03 in patients aged 60 or older. Intriguingly, the median AKIN_Cr_ remained consistently at 0 both before and after the prone position in both age groups. In contrast, the mean AKIN_UO_ showed an elevation in both patients under 60 and those over 60 years old (increasing from 0.62 to 0.96 and from 0.74 to 1.58, respectively). However, the median AKIN_UO_ significantly ascended from AKIN 0 to AKIN 2 after proning among patients aged 60 or older (median, *p* < 0.01), whereas among younger patients, AKIN_UO_ remained at 0 both before and after transitioning to proning. After returning patients to a supine position, there were no changes in either the median AKIN_Cr_ or AKIN_UO_ in either age group (Supplementary Materials: Tables S6 and S7).

### 3.5. Multivariable Logistic Regression

A multivariable logistic regression analysis was conducted to predict the occurrence of AKI (as defined by AKIN worsening) after prone positioning, as well as to adjust variables found to be statistically significant in the univariate analysis. The regression model included the patients’ age, lactate level, and position change. The prone position was found to be significantly associated with AKI development (odds ratio 2.38; 95% confidence interval, 1.05–5.36; *p* = 0.037). Patient age was also associated with AKI (odds ratio 1.04 for each year of life; 95% confidence interval, 11.02–1.07; *p* < 0.01), while lactate levels did not reach statistical significance (odds ratio 1.25; 95% confidence interval, 0.92–1.71; *p* = 0.2) (Table 4). A multivariate logistic regression stratified by COVID-19 infection was also conducted, and no significant differences were revealed in post-proning AKI by separating patients into COVID-19 vs. non-COVID-19 ARDS (Supplementary Materials: Table S9).

### 3.6. Time to AKI Post Prone

**Figure 2:**
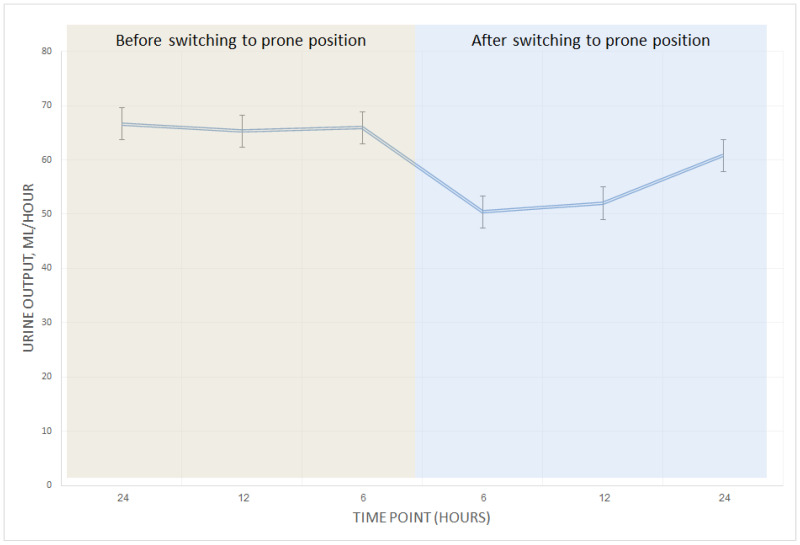
Average urine output in patients with overweight or obesity before and after prone position. Mean urine output per hour measured 24 h before and after prone position. The lowest urine output among overweight or obese patients was measured six hours post prone position (*p* < 0.01), then improved gradually. No significant change was observed among non-obese patients; thus, the chart only includes overweight or obese patients.

In the overall cohort, the average urine output per hour was lowest at 6 h following pronation (mean of 59 mL/h, *p*-value < 0.01 when compared to the mean urine output during the 6 hours before pronation), with subsequent gradual improvement (Figure 2). In the overall cohort, 42% (n = 36) of the cases who underwent a proning intervention event demonstrated AKI (as defined by worsening in AKIN criteria, creatinine or UO, by at least one point after prone position) within 24 h of being placed in the prone position. Among the cases where AKI was observed post proning, 55.5% (n = 20 out of 36) had a mean urine output of less than 0.5 milliliters per kilogram per hour during the 24 h post pronation. In 33.33% of these cases (n = 12), AKI was still present prior to the patient’s return to the supine position, emphasizing that the post-proning AKI is often not transient. A comparison of the AKIN score measured after proning and directly before returning the patients to the supine position showed a slight decrease in the mean AKIN_UO_ score but no change in the median AKIN_UO_ score (Table 5). No significant change was observed in the AKIN_Cr_ (Supplementary Materials: Table S10).

## 4. Discussion

Our main findings suggest that prone positioning in overweight or obese patients (BMI ≥ 25) treated for ARDS significantly increases the risk of developing AKI, as defined by the AKIN criteria [29]. This effect was observed exclusively in this population, while no similar impact was noted among non-obese patients, regardless of whether AKI was assessed via creatinine levels or urine output. Notably, returning patients to the supine position did not significantly affect AKI occurrence or renal function across any groups, with the exception of a minor rise in urea levels.

We observed that AKI incidence peaked six hours post prone maneuver, with gradual improvement in urine output thereafter. However, a high proportion of patients (55.55%) experienced AKI that persisted for up to 24 h following the prone intervention. Upon repositioning into the supine position, 33.33% of patients still displayed signs of AKI, accompanied by only a slight reduction in their AKIN_UO_ scores. These findings suggest that while renal function may begin to recover within hours, prone positioning might exert lasting effects on kidney function in some patients.

Not only did the incidence of AKI worsen following prone positioning among obese patients, but a near-significant reduction in the eGFR (*p* = 0.07) was also noted exclusively in this group, contrasting with non-obese prone patients where no such trend was observed. Nevertheless, clinicians often rely on AKI and creatinine rise as key markers, with the literature emphasizing creatinine worsening as a significant risk factor for mortality among ICU patients [16].

Patient age was also identified as a significant risk factor for the development of AKI, with a higher risk observed in patients aged 60 years or older. Our findings align with previous studies [34,35], which have shown that elderly patients with AKI face a greater risk of complications [36,37]. However, in multivariate analysis, we found that the prone position maneuver exerted a stronger influence on AKI development compared to age (odds ratio: 2.38 vs. 1.04 per year of life, respectively). Other variables, such as MAP, lactate levels, and SOFA scores, did not show significant changes in either univariate or multivariate analyses.

Prior to the current study, only a few investigations evaluated the negative impact of the prone position on renal function [25,26]. To date, no definitive conclusion has been drawn on whether prone positioning significantly increases the risk of AKI or whether returning to the supine position can effectively reverse renal impairment. Additionally, the relationship between BMI, AKI, and prone positioning has not been previously explored. Weig et al. examined the effects of abdominal obesity on organ function in patients with H1N1-associated ARDS undergoing prolonged cumulative prone positioning [26]. That study reported a higher incidence of renal failure in abdominally obese patients compared to non-abdominally obese ones (83% vs. 35%; *p* < 0.01), although abdominal obesity was defined by a sagittal abdominal diameter of ≥26 cm, without consideration of BMI.

Our findings reveal that prone positioning is a safe intervention for non-obese patients, with no significant impact on renal function. However, in overweight or obese patients, kidney failure can be expected within hours of prone positioning, potentially persisting for an extended period. Thus, careful preparation and monitoring are essential when managing these patients. In this context, our results support Weig et al.’s recommendation of exercising caution with prone positioning in obese patients [26].

The precise pathophysiology connecting prone positioning to renal failure remains incompletely understood. One proposed mechanism is the induction of intra-abdominal hypertension (IAH) by the prone position [24,38], which has been associated with impaired renal function due to elevated renal vein pressure, reduced arterial perfusion, and decreased glomerular filtration rate [39,40,41,42,43,44]. This effect is particularly significant in obese patients, who are more susceptible to IAH and its renal implications [40,45,46]. Additionally, increased renal parenchymal pressure in the prone position may lead to renal ischemia, further contributing to AKI [44]. Obesity exacerbates this risk, as it is associated with glomerular hypertrophy, which heightens the kidneys’ vulnerability to ischemic injury [22].

Urologic surgeries, particularly percutaneous nephrolithotomy (PCNL) performed in the prone position, have been associated with an increased risk of AKI [47,48,49,50,51]. This risk is attributed to factors such as hemodynamic changes, increased intra-abdominal pressure, and compartment syndrome during prolonged procedures [47,52]. To mitigate these risks, careful patient positioning, vigilant intraoperative monitoring, and minimizing operative time are essential strategies. Additionally, maintaining adequate hydration and ensuring proper padding can help reduce the incidence of AKI in patients undergoing urologic surgeries in the prone position [47,48].

Prone positioning is known to offer significant respiratory benefits [4,53,54,55,56], improving outcomes in patients with severe ARDS [57,58,59]. These patients often have the most critical form of ARDS (PaO_2_/FiO_2_ ratio < 100) and have exhausted all other treatment options. Therefore, avoiding prone positioning in critically ill patients to prevent AKI may not always be feasible. Nevertheless, AKI has been linked to poor outcomes and increased mortality, particularly in critically ill populations [16,18,60,61,62]. It remains important to understand the underlying causes of AKI and to explore preventative strategies.

Our findings raise the question of whether specific proning techniques might reduce the risk of AKI, particularly in obese patients. While only a few studies have addressed this issue [24,63], no prospective trial has systematically evaluated the impact of tailored proning techniques on renal outcomes. There is a pressing need for prospective studies that focus on optimizing prone positioning for obese patients to prevent renal deterioration without compromising the respiratory benefits of this intervention.

Given that AKI is a significant independent risk factor for mortality in ICU patients [18], and considering the increased risk of AKI associated with prone positioning in obese patients, a comprehensive prospective investigation is warranted. This need is even more urgent in the post-COVID-19 era, where obese patients experienced higher mortality rates during the pandemic. The increased susceptibility of obese individuals to future respiratory pandemics, combined with the higher mortality risk, is further complicated by the more frequent use of prone positioning in ICUs post COVID-19 compared to pre-pandemic levels. These factors suggest that the mortality risk could be amplified in future respiratory pandemics, underscoring the necessity of further research and preventative measures.

This study has several limitations. First, being a retrospective single-center study inherently restricts our sample size and the ability to establish causality. Second, because prone positioning is a validated treatment for hypoxemic ARDS patients, conducting a standard case–control study with a control group of similarly severe hypoxemic patients who did not receive prone positioning was not feasible. Consequently, we utilized a suboptimal pre–post study design, complicating the differentiation between the effects of prone positioning on kidney function and the patients’ overall clinical deterioration. While we collected data on potential confounders such as the MAP, SOFA score, and lactate levels pre and post proning, no significant changes were observed in these parameters, indicating that AKI may be more closely associated with the prone position itself than with systemic clinical decline. However, the complete elimination of inherent biases is unattainable.

Third, we did not directly measure intra-abdominal pressure or abdominal obesity, relying instead on BMI as a proxy for obesity, which limits our ability to definitively establish causality between these factors and AKI. Additionally, data were collected within 24 h of the position change, focusing on the immediate effects of prone positioning on kidney injury. Therefore, the longer-term impacts of prone positioning remain unclear. Another limitation is that the mean time in the prone position exceeded the recommended 16 h, as per the PROSEVA study. This was due to two factors: the slow implementation of the 16 h proning regimen and the increased patient load during the COVID-19 pandemic, which restricted treatment options and prolonged proning times. The question of whether shorter proning durations could reduce the incidence of prone-related AKI remains open.

## 5. Conclusions

In conclusion, our study highlights a significant association between prone positioning and the development of AKI in overweight or obese patients with ARDS. This may be due to the increased vulnerability of the kidneys to IAH. While prone positioning is an established intervention that offers substantial respiratory benefits in ARDS management, our findings indicate that it may pose an increased risk of renal impairment, particularly in this patient population. Given the higher morbidity associated with AKI and its potential long-term consequences, careful monitoring and consideration of alternative strategies may be warranted when implementing prone positioning in obese patients. Future research should focus on optimizing proning techniques and exploring preventive measures to mitigate renal risks without compromising respiratory outcomes, especially in the context of the ongoing challenges posed by increasing obesity rates and the lingering effects of the COVID-19 pandemic on ICU practices.

## Figures and Tables

**Table 1:** Baseline patient characteristics.

Characteristics	Total (N = 56)
Age	
Mean ± SD (N)	58 ± 15 (55)
Median (IQR)	59 (48, 70)
Range	22, 81
Sex	
Female	16/56 (29%)
Male	40/56 (71%)
Weight	
Mean ± SD (N)	93 ± 20 (55)
Median (IQR)	90 (80, 100)
Range	60, 150
Body mass index (BMI)	
Mean ± SD (N)	33 ± 7 (44)
Median (IQR)	31 (28, 35)
Range	20, 52
COVID-19 infection	39/56 (70%)
Baseline creatinine, mg/dL	
Mean ± SD (N)	0.79 ± 0.22 (55)
Median (IQR)	0.80 (0.65, 0.89)
Range	0.31, 1.40
PaO_2_/FiO_2_	
Mean ± SD (N)	74 ± 44 (50)
Median (IQR)	64 (57, 77)
Range	38, 324
SOFA pre prone	
Mean ± SD (N)	7.84 ± 2.66 (56)
Median (IQR)	8.00 (5.75, 9.00)
Range	1.00, 14.00

**Table 2:** Univariate analysis of AKIN criteria based on creatinine (mg/dL), stratified by BMI.

		Prone			Supine		
		AKIN Prior to Pronation (Event/Base)	AKIN After Pronation (24 h/Base)	*p*-Value	AKIN Prior to Supination (Event/Base)	AKIN After Supination (24 h/Base)	*p*-Value
All Patients		Prone (N = 86 cases, 56 patients)			Supine (N = 59 cases, 39 patients)		
	Mean ± SD (N)	0.53 ± 0.97 (85)	0.93 ± 1.16 (81)	<0.01	0.59 ± 1.07 (59)	0.71 ± 1.17 (55)	0.07
	Median (IQR)	0.00 (0.00, 1.00)	0.00 (0.00, 2.00)		0.00 (0.00, 1.00)	0.00 (0.00, 1.00)	
	Range	0.00, 3.00	0.00, 3.00		0.00, 3.00	0.00, 3.00	
Patients with BMI < 25		Prone (N = 11 cases, 5 patients)			Supine (N = 11 cases, 4 patients)		
	Mean ± SD (N)	0.09 ± 0.30 (11)	0.13 ± 0.35 (8)	NA	0.00 ± 0.00 (11)	0.00 ± 0.00 (9)	NA
	Median (IQR)	0.00 (0.00, 0.00)	0.00 (0.00, 0.00)		0.00 (0.00, 0.00)	0.00 (0.00, 0.00)	
	Range	0.00, 1.00	0.00, 1.00		0.00, 0.00	0.00, 0.00	
Patients with BMI ≥ 25		Prone (N = 57 cases, 39 patients)			(Supine N = 37 cases, 17 patients)		
	Mean ± SD (N)	0.58 ± 0.96 (57)	0.96 ± 1.10 (57)	<0.01	0.76 ± 1.14 (37)	0.95 ± 1.27 (37)	0.07
	Median (IQR)	0.00 (0.00, 1.00)	1.00 (0.00, 2.00)		0.00 (0.00, 2.00)	0.00 (0.00, 2.00)	
	Range	0.00, 3.00	0.00, 3.00		0.00, 3.00	0.00, 3.00	

Univariate analysis compares Acute Kidney Injury Network (AKIN) scores based on creatinine (mg/dL) levels measured before and after changes in patient position (prone or supine). The table presents the AKIN scores for all patients, along with subdivisions by body mass index (BMI). The AKIN scores are calculated as follows: Event/Base: the AKIN score is determined by comparing the patient’s creatinine level before the position change (prone or supine) to their baseline creatinine level; 24/Base: the AKIN score is calculated by comparing the creatinine level measured within 24 h after the position change to the patient’s baseline creatinine level.

**Table 3:** Univariate analysis of AKIN criteria based on urine output (mL/h), stratified by BMI.

		Prone			Supine		
		AKIN Prior to Pronation	AKIN After Pronation	*p*-Value	AKIN Prior to Supination	AKIN After Supination	*p*-Value
All Patients		Prone (N = 86 cases, 56 patients)			Supine (N = 59 cases, 39 patients)		
	Mean ± SD (N)	0.67 ± 1.07 (85)	1.22 ± 1.28 (86)	<0.01	0.93 ± 1.17 (59)	0.98 ± 1.20 (59)	0.62
	Median (IQR)	0.00 (0.00, 1.00)	1.00 (0.00, 2.00)		0.00 (0.00, 2.00)	0.00 (0.00, 2.00)	
	Range	0.00, 3.00	0.00, 3.00		0.00, 3.00	0.00, 3.00	
Patients with BMI < 25		Prone (N = 11 cases, 5 patients)			Supine (N = 11 cases, 4 patients)		
	Mean ± SD (N)	0.09 ± 0.30 (11)	0.45 ± 1.04 (11)	0.34	0.18 ± 0.60 (11)	0.45 ± 0.82 (11)	0.37
	Median (IQR)	0.00 (0.00, 0.00)	0.00 (0.00, 0.00)		0.00 (0.00, 0.00)	0.00 (0.00, 0.50)	
	Range	0.00, 1.00	0.00, 3.00		0.00, 2.00	0.00, 2.00	
Patients with BMI ≥ 25		Prone (N = 57 cases, 39 patients)			Supine (N = 37 cases, 17 patients)		
	Mean ± SD (N)	0.84 ± 1.18 (57)	1.35 ± 1.26 (57)	<0.01	1.27 ± 1.17 (37)	1.32 ± 1.27 (37)	0.74
	Median (IQR)	0.00 (0.00, 2.00)	2.00 (0.00, 2.00)		2.00 (0.00, 2.00)	1.00 (0.00, 3.00)	
	Range	0.00, 3.00	0.00, 3.00		0.00, 3.00	0.00, 3.00	

Univariate analysis compares Acute Kidney Injury Network (AKIN) scores based on urine output (mL/h) measured before and after changes in patient position (prone or supine). The table presents the AKIN scores for all patients, along with subdivisions by body mass index (BMI).

**Table 4:** Multivariate logistic regression model for predicting AKI occurrence after prone position.

Characteristic	OR	95% CI	*p*-Value
Change in patient position to prone position	2.38	1.05, 5.36	0.037
Age	1.04	1.02, 1.07	<0.001
Lactate difference	1.25	0.92, 1.71	0.2

A multivariable logistic regression model predicting Acute Kidney Injury Network (AKIN) criteria worsening (based on creatinine or urine output) as the main independent variable. Variables found to be statistically significant in the univariate analysis were also included in the regression model. AKI, acute kidney injury; CI, confidence interval; OR, odds ratio.

**Table 5:** Univariate analysis of AKIN scores based on urine output (mL/h) at different time points in patients with AKI after prone positioning.

Time Points		Before Prone Position (N = 36)	After Prone Position (N = 36)	Before Supine Position (N = 21)	*p*-Value ^1^ Before vs. After Prone	*p*-Value ^2^ (After Prone vs. Before Supine)
Patients with AKI development	Mean ± SD (N)	0.72 ± 1.03 (36)	2.14 ± 0.96 (36)	1.33 ± 1.35 (21)	<0.01	<0.01
	Median (IQR)	0.00 (0.00, 1.25)	2.00 (2.00, 3.00)	2.00 (0.00, 3.00)		
	Range	0.00, 3.00	0.00, 3.00	0.00, 3.00		

Univariate analysis compares Acute Kidney Injury Network (AKIN) scores measured at three key time points: (1) AKIN before patients were placed in the prone position; (2) AKIN after prone positioning, representing the worst AKIN score observed within 24 h following proning; and (3) AKIN measured directly before patients were returned to the supine position. The table shows AKIN scores based on urine output (measured in mL/h). The analysis was conducted only on patients who developed AKI after prone positioning. Additionally, the table presents the *p*-values for two statistical comparisons: ^1^ the *p*-value for comparison between AKIN before and after prone positioning and the ^2^ *p*-value for comparison between AKIN after prone positioning and AKIN measured just before returning to the supine position.

## Data Availability

The data presented in this study are available on request from the corresponding author.

## Supplementary Materials

The following supporting information can be downloaded at https://www.mdpi.com/article/10.3390/jcm14020631/s1: Table S1: Baseline patient comorbidities; Table S2: Univariate analysis of lab results during ICU admission: before and after prone and supine position; Table S3: Univariate analysis of patients’ SOFA scores during ICU admission, stratified by BMI; Table S4: Univariate analysis of patients’ urea and creatinine during ICU admission: before and after prone and supine position; Table S5: Analysis of patient BMI; Table S6: Univariate analysis of AKIN criteria based on creatinine (mg/dL), stratified by age; Table S7: Univariate analysis of AKIN criteria based on urine output (mL/h), stratified by age; Table S8: Univariate analysis of eGFR before and after prone position, stratified by BMI; Table S9: Multivariate logistic regression model for predicting AKI occurrence, stratified by COVID-19 infection; and Table S10: Univariate analysis of AKIN scores based on creatinine (mg/dL) at different time points in patients with AKI after prone positioning.
