# Supplementary material for: Assessing the Impact of the Prone Position on Acute Kidney Injury
Source: J Clin Med. 2025 Jan 19;14(2):631. doi: 10.3390/jcm14020631 (PMC11766097; doi:10.3390/jcm14020631)
Supplement: Supplementary file 1 [file jcm-14-00631-s001.zip › jcm-3383170-supplementary.pdf]

Journal of
Clinical Medicine

MDPI

Supplementary Materials:

Table S1. Baseline patient comorbidities.

Comorbidities	Total (N = 56)
Diabetes mellitus type 2	19 / 56 (34%)
Hypertension	22 / 56 (39%)
Congestive heart failure	2 / 56 (3.6%)
Malignancy	1 / 56 (1.8%)
Malignancy - Glioblastoma	1 / 56 (1.8%)
Breast cancer	1 / 56 (1.8%)
Nephrotic syndrome	1 / 56 (1.8%)
Chronic renal failure	1 / 56 (1.8%)
Basal cell carcinoma	1 / 56 (1.8%)
Hydronephrosis	1 / 56 (1.8%)

Table S2. Univariate analysis of lab results during ICU admission: before and after prone and supine position.

	Prone (N = 86 cases, 56 patients)			Supine (N = 59 cases, 39 patients)		
Characteristic	Prior to Pronation	Worst Level within 24 Hours after Pronation	P-value	Prior to Supination	Worst Level within 24 Hours after Supination	P-value
Serum Hemoglobin, g/dL			<0.01			<0.01
Mean ± SD (N)	10.91 ± 2.53 (84)	10.64 ± 2.57 (86)		10.29 ± 2.06 (59)	9.70 ± 1.86 (59)	
Median (IQR)	10.30 (8.88, 13.30)	10.15 (8.50, 12.40)		10.30 (8.60, 11.65)	9.40 (8.20, 11.15)	
Range	6.70, 17.00	6.80, 17.40		7.10, 16.10	6.60, 14.80	
Platelets, 10^3u/L			0.26			0.05
Mean ± SD (N)	291 ± 126 (84)	301 ± 148 (86)		278 ± 125 (59)	266 ± 131 (59)	
Median (IQR)	284 (214, 358)	274 (204, 392)		270 (186, 357)	250 (169, 343)	
Range	61, 691	61, 753		21, 592	21, 577	
Bilirubin (Total), mg/dL			0.06			0.63
Mean ± SD (N)	0.53 ± 0.58 (73)	0.77 ± 0.93 (65)		0.66 ± 0.84 (47)	0.56 ± 0.99 (45)	
Median (IQR)	0.00 (0.00, 1.00)	1.00 (0.00, 1.00)		1.00 (0.00, 1.00)	0.00 (0.00, 1.00)	
Range	0.00, 2.00	0.00, 5.00		0.00, 4.00	0.00, 6.00	
Creatine phosphokinase, IU/L			0.07			0.01
Mean ± SD (N)	154 ± 387 (47)	325 ± 1,090 (55)		368 ± 587 (38)	476 ± 673 (35)	
Median (IQR)	65 (35, 143)	73 (44, 206)		112 (40, 408)	204 (59, 560)	
Range	10, 2,628	12, 7,975		11, 3,034	12, 3,304	
PH			<0.01			0.01
Mean ± SD (N)	7.31 ± 0.09 (85)	7.27 ± 0.14 (86)		7.42 ± 0.09 (59)	7.38 ± 0.15 (59)	
Median (IQR)	7.33 (7.26, 7.37)	7.27 (7.17, 7.33)		7.43 (7.36, 7.49)	7.39 (7.28, 7.48)	
Range	7.04, 7.50	6.99, 7.61		7.22, 7.61	7.00, 7.71	
Serum lactate, mmol/L			<0.01			<0.01
Mean ± SD (N)	2.17 ± 1.35 (85)	3.24 ± 2.69 (86)		1.78 ± 0.86 (59)	2.64 ± 1.75 (59)	
Median (IQR)	2.10 (1.40, 2.60)	2.45 (1.70, 3.70)		1.80 (1.05, 2.20)	2.40 (1.60, 2.95)	
Range	0.00, 9.50	1.00, 14.10		0.40, 5.30	0.80, 11.80	

Table S3. Univariate analysis of patient's SOFA score during ICU admission, stratified by BMI.

		**Prone**			**Supine**		
		**Prior to Pronation**	**24 Hours after Pronation**	**P-value**	**Prior to Supination**	**24 Hours after Supination**	**P-value**
		Prone (N = 86 cases, 56 patients)			Supine (N = 59 cases, 39 patients)		
All Patients	Mean ± SD (N)	7.91 ± 2.51 (86)	8.15 ± 2.71 (86)	0.08	6.68 ± 2.71 (59)	6.92 ± 2.79 (59)	0.07
	Median (IQR)	8.00 (6.00, 9.00)	8.50 (6.00, 9.75)		7.00 (5.00, 8.00)	7.00 (5.00, 8.50)	
	Range	1.00, 14.00	1.00, 14.00		1.00, 13.00	1.00, 13.00	
		Prone (N = 11 cases, 5 patients)			Supine (N = 11 cases, 4 patients)		
Patients with BMI < 25	Mean ± SD (N)	8.73 ± 0.79 (11)	8.64 ± 1.12 (11)	1	7.82 ± 0.40 (11)	8.00 ± 0.45 (11)	0.34
	Median (IQR)	9.00 (8.50, 9.00)	9.00 (8.00, 9.00)		8.00 (8.00, 8.00)	8.00 (8.00, 8.00)	
	Range	7.00, 10.00	7.00, 11.00		7.00, 8.00	7.00, 9.00	
		Prone (N = 57 cases, 39 patients)			Supine (N = 37 cases, 17 patients)		
Patients with BMI ≥ 25	Mean ± SD (N)	7.67 ± 2.54 (57)	7.93 ± 2.82 (57)	0.1	6.4 ± 3.0 (37)	6.7 ± 3.1 (37)	0.12
	Median (IQR)	8.00 (5.00, 9.00)	8.00 (5.00, 10.00)		6.0 (5.0, 8.0)	7.0 (4.0, 9.0)	
	Range	1.00, 14.00	1.00, 14.00		1.0, 13.0	1.0, 13.0	

ICU intensive care unit; SOFA sequential organ failure assessment.

Table S4. Univariate analysis of patient's urea and creatinine during ICU admission: before and after prone and supine position.

	**Prone – (N = 86 cases, 56 patients)**			**Supine – (N = 59 cases, 39 patients)**		
**Characteristic**	**Prior to Pronation**	**Highest Level within 24 Hours after Pronation**	**P-value**	**Prior to Supination**	**Highest Level within 24 Hours after Supination**	**P-value**
Serum UREA, mg/dL			<0.01			<0.01
Mean ± SD (N)	73 ± 55 (85)	86 ± 61 (86)		79 ± 68 (59)	86 ± 68 (58)	
Median (IQR)	60 (34, 89)	74 (43, 110)		62 (32, 107)	70 (34, 129)	
Range	11, 279	8, 286		8, 378	8, 329	
Serum creatinine, mg/dL			<0.01			0.11
Mean ± SD (N)	0.98 ± 1.01 (85)	1.26 ± 1.31 (85)		1.09 ± 1.50 (59)	1.10 ± 1.35 (58)	
Median (IQR)	0.70 (0.40, 1.20)	0.80 (0.50, 1.60)		0.60 (0.40, 1.05)	0.60 (0.30, 1.18)	
Range	0.00, 6.10	0.00, 6.80		0.00, 8.20	0.00, 6.70	

Table S5. Analysis of patient BMI.

	All Patients	Patients with BMI ≥ 25	Patients with BMI < 25
Mean ± SD (N)	33 ± 7 (44)	34 ± 7 (39)	23.27 ± 1.82 (5)
Median (IQR)	31 (28, 35)	33 (28, 36)	24.22 (22.49, 24.22)
Range	20, 52	26, 52	20.45, 24.97

Patients were divided into two groups: overweight or obese (BMI ≥ 25) and non-obese (BMI < 25). BMI was calculated by height and weight as recorded in the MetaVision system during ICU admission, or as recorded in the Chameleon system prior to ICU admission. BMI body mass index; ICU intensive care unit.

Table S6. Univariate analysis of AKIN criteria based on creatinine (mg/dL), stratified by age.

		Prone			Supine		
		AKIN Prior Pronation (event/base)	AKIN After Pronation (24h/base)	P-value	AKIN Prior Supination (event/base)	AKIN After Supination (24h/base)	P-value
All Patients		Prone (N = 86 cases, 56 patients)			Supine (N = 59 cases, 39 patients)		
	Mean ± SD (N)	0.53 ± 0.97 (85)	0.93 ± 1.16 (81)	<0.01	0.59 ± 1.07 (59)	0.71 ± 1.17 (55)	0.07
	Median (IQR)	0.00 (0.00, 1.00)	0.00 (0.00, 2.00)		0.00 (0.00, 1.00)	0.00 (0.00, 1.00)	
	Range	0.00, 3.00	0.00, 3.00		0.00, 3.00	0.00, 3.00	
Age < 60		Prone (N = 50 cases)			Supine (N = 37 cases)		
	Mean ± SD (N)	0.46 ± 0.93 (50)	0.85 ± 1.11 (46)	<0.01	0.46 ± 0.93 (37)	0.60 ± 1.12 (35)	0.34
	Median (IQR)	0.00 (0.00, 0.00)	0.00 (0.00, 1.00)		0.00 (0.00, 0.00)	0.00 (0.00, 0.50)	
	Range	0.00, 3.00	0.00, 3.00		0.00, 3.00	0.00, 3.00	
Age ≥ 60		Prone (N = 36 cases)			Supine (N= 22 cases)		
	Mean ± SD (N)	0.63 ± 1.03 (35)	1.03 ± 1.22 (35)	<0.01	0.82 ± 1.26 (22)	0.90 ± 1.25 (20)	0.15
	Median (IQR)	0.00 (0.00, 1.00)	0.00 (0.00, 2.00)		0.00 (0.00, 2.00)	0.00 (0.00, 2.00)	
	Range	0.00, 3.00	0.00, 3.00		0.00, 3.00	0.00, 3.00	

Univariate analysis compares Acute Kidney Injury Network (AKIN) scores based on creatinine (mg/dL) levels measured before and after changes in patient position (prone or supine). The table presents the AKIN scores for all patients, along with subdivisions by age group. The AKIN scores are calculated as follows: Event/Base: The AKIN score is determined by comparing the patient's creatinine level before the position change (prone or supine) to their baseline creatinine level. 24/Base: The AKIN score is calculated by comparing creatinine level measured within 24 hours after the position change, to the patient's baseline creatinine level.

Table S7. Univariate analysis of AKIN criteria based on urine output (ml/hour), stratified by age.

		Prone			Supine		
		**AKIN Prior Pronation**	**AKIN After Pronation**	**P-value**	**AKIN Prior Supination**	**AKIN After Supination**	**P-value**
		Prone (N = 86 cases, 56 patients)			Supine (N = 59 cases, 39 patients)		
All Patients	Mean ± SD (N)	0.67 ± 1.07 (85)	1.22 ± 1.28 (86)	<0.01	0.93 ± 1.17 (59)	0.98 ± 1.20 (59)	0.62
	Median (IQR)	0.00 (0.00, 1.00)	1.00 (0.00, 2.00)		0.00 (0.00, 2.00)	0.00 (0.00, 2.00)	
	Range	0.00, 3.00	0.00, 3.00		0.00, 3.00	0.00, 3.00	
		Prone (N = 50 cases)			Supine (N = 37 cases)		
Age < 60	Mean ± SD (N)	0.62 ± 1.03 (50)	0.96 ± 1.26 (50)	0.02	0.62 ± 1.06 (37)	0.78 ± 1.16 (37)	0.19
	Median (IQR)	0.00 (0.00, 1.00)	0.00 (0.00, 2.00)		0.00 (0.00, 2.00)	0.00 (0.00, 2.00)	
	Range	0.00, 3.00	0.00, 3.00		0.00, 3.00	0.00, 3.00	
		Prone (N = 36 cases)			Supine (N = 22 cases)		
Age ≥ 60	Mean ± SD (N)	0.74 ± 1.15 (35)	1.58 ± 1.23 (36)	<0.01	1.45 ± 1.18 (22)	1.32 ± 1.21 (22)	0.52
	Median (IQR)	0.00 (0.00, 1.50)	2.00 (0.00, 3.00)		2.00 (0.00, 2.00)	1.00 (0.00, 2.00)	
	Range	0.00, 3.00	0.00, 3.00		0.00, 3.00	0.00, 3.00	

Univariate analysis compares Acute Kidney Injury Network (AKIN) scores based on urine output (ml/hour) measured before and after changes in patient position (prone or supine). The table presents the AKIN scores for all patients, along with subdivisions by age group.

Table S8. Univariate analysis of eGFR before and after prone position, stratified by BMI.

		**eGFR Prior Pronation**	**eGFR After Pronation**	**P-value**
		Prone (N = 86 cases, 56 patients)		
All Patients	Mean ± SD (N)	182± 190 (65)	167± 194 (64)	0.07
	Median (IQR)	143 (90, 184)	124 (54, 175)	
	Range	22, 917	14, 917	
		Prone (N = 11 cases, 5 patients)		
Patients with BMI < 25	Mean ± SD (N)	484 ± 378 (8)	476 ± 420 (7)	0.85
	Median (IQR)	384 (157, 917)	309 (108, 917)	
	Range	43, 917	35, 917	
		Prone (N = 57 cases, 39 patients)		
Patients with BMI ≥ 25	Mean ± SD (N)	139 ± 92 (57)	129 ± 101 (57)	0.07
	Median (IQR)	126 (76,172)	120 (51, 170)	
	Range	22, 548	14, 548	

Univariate analysis compares estimated glomerular filtration rate (eGFR) before and after prone position. The table presents the eGFR measurements for all patients, along with subdivisions by body mass index (BMI).

Table S9. Multivariate logistic regression model for predicting AKI occurrence, stratified by COVID-19 infection.

	Covid-19 Infection			Non-Covid-19 Infection		
**Characteristic**	**OR1**	**95% CI**	**p-value**	**OR1**	**95% CI**	**P-value**
Change of Patient Position to Prone Position	2.52	1.11, 5.72	0.027	2.47	0.52, 11.8	0.3

A multivariable logistic regression model predicting Acute Kidney Injury Network (AKIN) criteria worsening (based on creatinine or urine output) as the main independent variable, stratified by COVID-19 infection diagnosis. AKI acute kidney injury; CI Confidence Interval; OR Odds Ratio.

Table S10. Univariate analysis of AKIN scores based on creatinine (mg/dL) at different time points in patients with AKI after prone positioning.

**Time Points**		**Before Prone Position (N= 36)**	**After Prone Posion (N= 36)**	**Before Supine Position (N= 21)**	**P-value[1] Before vs. After Prone**	**P-value[2] (After Prone vs. Before Supine)**
Patients with AKI Development	Mean ± SD (N)	0.53 ± 0.94 (36)	1.37 ± 1.11 (35)	0.90 ± 1.26 (21)	<0.01	0.21
	Median (IQR)	0.00 (0.00, 1.00)	1.00 (0.50, 2.00)	0.00 (0.00, 2.00)		
	Range	0.00, 3.00	0.00, 3.00	0.00, 3.00		

Univariate analysis compares Acute Kidney Injury Network (AKIN) scores measured at three key time points: **(1)** AKIN before patients were placed in the prone position; **(2)** AKIN after prone positioning, representing the worst AKIN score observed within 24 hours following proning; and **(3)** AKIN measured directly before patients were returned to the supine position. The table shows AKIN scores based on creatinine (measured in mg/dL). The analysis was conducted only on patients who developed AKI after prone positioning. Additionally, the table presents the **p-values for two statistical comparisons:** [1]the p-value for comparison between AKIN before and after prone positioning, [2]p-value for comparison between AKIN after prone positioning and AKIN measured just before returning to the supine position.
